# Zoonotic disease outbreaks reported under India's **I**ntegrated **D**isease **S**urveillance **P**rogramme, 2018–2023: a cross-sectional analysis of national surveillance data

**DOI:** 10.1016/j.lansea.2025.100601

**Published:** 2025-05-20

**Authors:** Mogan Kaviprawin, Mohankumar Raju, Manikandanesan Sakthivel, Archana Ramalingam

**Affiliations:** a Field Epidemiology Training Program, Indian Council of Medical Research - National Institute of Epidemiology, Chennai, India; b South Asia Field Epidemiology and Technology Network, Inc., (SAFETYNET), India; c South Asia Field Epidemiology and Technology Network, Inc., (SAFETYNET), Philippines

**Keywords:** Disease outbreaks, Zoonoses, Public health surveillance, Epidemiology, India

## Abstract

**Background:**

Timely analysis of zoonotic outbreak surveillance data is critical for assessing the effectiveness of outbreak detection and reporting systems, a priority for global health security. We described the zoonotic disease outbreaks notified under the Integrated Disease Surveillance Program (IDSP) in India between 2018 and 2023 to identify temporal trends and spatial variation.

**Methods:**

We conducted a cross-sectional study by reviewing zoonotic disease outbreak line-list data from IDSP weekly outbreak reports and analyzed by year, region, and timeliness of reporting. We conducted a mixed Poisson regression to estimate the change (β coefficient) in outbreaks over the years and visualized maps in R software.

**Findings:**

Of the 6948 outbreaks reported in IDSP, 583 (8.3%) were zoonotic, with a median of seven monthly zoonotic outbreaks. Outbreaks significantly increased over the years (β coefficient = 0.07 [0.02–0.12]). Japanese encephalitis accounted for 29.5% of zoonotic outbreaks, followed by leptospirosis (18.7%) and scrub typhus (13.9%). The northeast region contributed 35.8% of zoonotic disease outbreaks, followed by the southern (31.7%) and western regions (15.4%). One-third (34.6%) of outbreaks were reported late, and they declined over the years (52.6% in 2019, 40.9% in 2021, and 5.2% in 2023). The follow-up reports were unavailable for 97.2% of zoonotic outbreaks notified.

**Interpretation:**

We documented the regions with high notification of zoonotic disease outbreaks under India's national-level outbreak surveillance system. Critical gaps in weekly outbreak reports were identified, particularly the lack of follow-up documentation. To address these gaps, we recommend strengthening disease-specific surveillance systems in hotspot regions.

**Funding:**

The present study is non-funded.

**Research in context**

**Evidence before this study**

We searched PubMed and MEDLINE for articles published from January 1, 2004 to March 10, 2025, using the keywords ((Zoonotic disease outbreak OR zoonoses outbreak) AND India AND Epidemiology). We identified 563 studies, with the majority documenting individual outbreak investigations. Though outbreaks of diseases including measles, chickenpox, and dengue were analyzed, a comprehensive analysis of all zoonotic disease outbreaks from the national-level infectious disease surveillance system had not been conducted. The lack of analysis of geographical patterns and documentation of reporting delays hindered evidence-based decision-making and targeted interventions.

**Added value of this study**

We analyzed the geographical patterns of 19 zoonotic disease outbreaks and identified disease-specific hotspots across India. We documented a significant increase in outbreaks over the past six years, specifically the post-COVID-19 pandemic. The study revealed critical operational gaps in the surveillance system, including delayed outbreak reporting and the absence of follow-up reports. The majority (94.2%) of the high-cluster districts in 2022–23 were concentrated in two regions of India (Southern and Northeast).

**Implications of all the available evidence**

We recommend incorporating the outbreak onset and reporting dates for the delayed outbreaks to accurately estimate delays in days and enhance the surveillance system at state and regional levels. The identified hotspots of zoonotic outbreaks guide the targeted strengthening of surveillance systems and control measures. A One Health approach, involving intersectoral coordination between human, animal, and environmental health sectors, is essential to improve early detection, timely response, and overall outbreak management.

## Introduction

Outbreaks and widespread disease events are frequently driven by emerging and reemerging diseases, with around two-thirds originating from zoonotic sources globally.^1^ Human activities, alongside environmental changes, intensify the likelihood of zoonotic diseases as they increase close contact between animals and humans.^2^ Zoonotic diseases present a global concern, yet they hold particular significance in low and low-middle-income countries (LMICs). LMICs documented human activities, including habitat encroachment and deforestation, intensify interactions between humans and wildlife, increasing the likelihood of zoonotic spillover.^3^^,^^4^

Timely analysis of zoonotic outbreak surveillance data was identified as a global research priority to assess the scope and effectiveness of outbreak detection and reporting.^5^ Enhancing the surveillance system has been a core focus of India's national “One Health” program.^6^ Disease alerts and outbreaks in India are documented and responded through the Integrated Disease Surveillance Program (IDSP).^7^ Surveillance data aids in early outbreak detection, enabling timely responses to prevent widespread transmission.^8^ By identifying region-specific trends and high-risk zones through surveillance data analysis, more efficient resource allocation could be facilitated, especially where public health resources are constrained.

The zoonotic diseases prioritized in India as of 2020 include Influenza, Anthrax, Japanese Encephalitis (JE), Leptospirosis, Brucellosis, Rabies, Scrub Typhus, and Crimean-Congo Haemorrhagic Fever (CCHF).^9^ Examining the Public Health Emergencies of International Concern such as the H1N1, Ebola, and Zika outbreaks revealed several factors that might influence delays in detecting outbreaks.^10^^,^^11^ India's infectious disease surveillance system in 2020 documented that one-third of events were reported delayed.^12^ However, a comprehensive analysis of the delayed notification of outbreaks at the national level has not been documented yet. Given India's pivotal role in global health security, strengthened surveillance aids regional and international preparedness and contribute to pandemic readiness.^13^

Under the outbreak surveillance system, a detailed analysis was conducted for diseases including chikungunya,^14^ dengue,^14^ measles,^15^ and zika virus,^16^ however a comprehensive analysis of zoonotic diseases was not available. Analyzing the compiled IDSP outbreak reports can help in identifying the gaps and describe potential limitations in documentation. There is a limited systematically conducted analysis on zoonotic disease to understand the impact of the COVID-19 pandemic on reporting. In our analysis, we assessed the pattern of zoonotic disease outbreaks during the COVID-19 pandemic and compared them before and after the pandemic. We described the zoonotic disease outbreaks notified under IDSP, India, by year, disease, and geographical regions. In addition, we estimated the case-fatality ratio and epidemiologically described the zoonotic outbreaks reported late from 2018 to 2023.

## Methods

### Study design, period, and data source

We conducted a cross-sectional study analyzing the outbreak data reported in the IDSP weekly outbreak dashboard from January 2018 to December 2023. We abstracted the outbreak or event alert reports published weekly in IDSP.^17^ IDSP captures the outbreaks from six regions (northern, northeastern, eastern, western, central, and southern) of India (Supplementary Table S1).^18^ India is divided into 28 states and 8 Union Territories. These states and territories are further subdivided into districts, which are administrative divisions designed for local governance and management.

### Reporting of outbreaks in IDSP

IDSP follows a decentralized approach, with case-based and event-based surveillance. Outbreaks or event alerts are captured through event-based surveillance.^7^ Community health workers capture unusual events such as clusters of illness, deaths, or rumors of outbreaks. The information is compiled and passed on to the Primary Health Center (PHC) and Community Health Center (CHC). Data are sent to the District Surveillance Officer (DSO) at the District Surveillance Unit (DSU). If an outbreak is confirmed, the DSO organizes a rapid response team (RRT) for investigation and response. RRTs conduct epidemiological investigations, implement control measures, and provide immediate public health interventions. The key response strategies include confirmation of outbreak, verification of diagnosis, drafting case definition following trawling interviews, active case search, and framing hypothesis based on the descriptive epidemiology. Additionally, investigation is continued to test hypothesis using analytical epidemiology and environmental investigations. Systematically, the recommendations were given to the authorities and the stakeholders to undertake the actions.^7^

The outbreaks generated are compiled at the district level and sent to the State Surveillance Unit (SSU) and then to the Central Surveillance Unit (CSU). The IDSP weekly outbreak reports are georeferenced at the district level. Point location data (latitude or longitude) is not systematically available in the IDSP weekly outbreak system dashboard. The primary reporting unit is the DSU, which aggregates data from health facilities and block-level reports.^19^ To prevent duplication of the same outbreaks from multiple units, authorities in the central level review, compile, and provide a single outbreak with multi-district or state transmission in the weekly report. Under IDSP, data on epidemic-prone diseases is collected weekly from Monday to Sunday. Reports are considered timely if they are submitted within one week after the last date of the reporting week. This means that for a reporting week ending on a Sunday, the data should be submitted by the following Sunday. The timely reporting is achieved when data for a given week is submitted within seven days after its completion. If the data is submitted beyond this seven-day grace period, it is considered late reporting.^19^^,^^20^

With the implementation of the real-time Integrated Health Information Platform (IHIP) as an information system under IDSP in 2021, event alerts generated at any field level are immediately reflected in the system, making them visible to all stakeholders, including MOs, DSUs, SSUs, and the CSU.^7^^,^^21^ The National Centre for Disease Control (NCDC), under the Ministry of Health and Family Welfare, India, manages the CSU.^19^ CSU compiles data from all SSUs across India and publishes weekly outbreak or event alert reports on the IDSP website. Though IHIP was implemented in 2021 to capture real-time cases, the outbreak report is published in IDSP in a weekly manner. Diseases reported under IDSP include Acute Diarrheal Disease (including acute gastroenteritis), Bacillary Dysentery, Viral Hepatitis, Enteric Fever, Malaria, Dengue, Chikungunya, Acute Encephalitis Syndrome, Meningitis, Measles, Diphtheria, Pertussis, Chicken Pox, Fever of unknown origin, Acute Respiratory Infection (ARI)/Influenza Like Illness (ILI), Pneumonia, Leptospirosis, Acute Flaccid Paralysis (<15 years of age), Rabies, Snake bite, Unusual Syndromes not captured by listed conditions and state specific diseases.^22^

### Zoonotic diseases included in the analysis

We included zoonotic disease outbreaks reported under IDSP weekly outbreak system including Anthrax, Brucellosis, CCHF, Rabies, Hand Foot and Mouth disease, JE, Leptospirosis, Scrub typhus, Kala-azar, Melioidosis, Nipah, Norovirus, Monkeypox, Kyasanur Forest Disease (KFD), Influenza (Avian or swine), Snakebite, Trypanosomiasis, West Nile fever, and Zika in the analysis.^23^, ^24^, ^25^

### Data abstraction process

In IDSP, reported outbreaks are merged for each week and published.^17^ Each week's data is downloaded in Portable Document Format (PDF) from the dashboard, converted to Microsoft Excel sheets, and merged subsequently. In the process of conversion to Excel sheets, there is a possibility of formatting errors due to variations in report structures and table formats, leading to potential loss of granular information. However, to prevent the errors, we have conducted validation checks such as cross-verification with original reports, consistency checks for missing values, and outliers. The key variables in the weekly outbreak report include the name of the state and district, the disease name, the number of cases and deaths, brief description of the actions taken, type of report (late or follow-up).^17^

### Data analysis

We distributed the outbreaks by year, state, district, and disease type. We summarized the quantitative variables as median with the Interquartile range (IQR). We estimated the case fatality ratio (CFR per 100 cases) by dividing the number of deaths by the cases. We categorized the timeline into three periods to analyze changes in outbreak patterns over the COVID-19 pandemic, i.e., pre-COVID period (2018–19), COVID period (2020–21), and post-COVID period (2022–23). We used mixed Poisson regression, adjusting for the states to estimate the change in reporting of zoonotic disease outbreaks between the categorized COVID periods. By conducting a sub-group analysis, we calculated the regression coefficient (β) to quantify the change in notification of zoonotic outbreaks between the categorized periods. Similarly, we estimated the regression coefficient (β) with a 95% confidence interval (CI) to infer the change in the number of outbreaks over the years, adjusting for states. We assessed whether there was spatial autocorrelation (clustering) of zoonotic disease outbreaks at the district level for each year using global Moran's I statistic. We used the queen contiguity method to map the neighborhoods for each district. In the queen contiguity method, the polygons that share a border or a corner are considered neighbors. We then used the Local Indicator of Spatial Association (LISA) to disaggregate the global Moran's index and identify the specific districts where there was spatial autocorrelation.^26^ In this analysis, each district was classified into one of the four LISA quadrants as follows, based on LISA value and p-value: High-High (HH), Low-Low (LL), Low-High (LH), and High-Low (HL). The HH and LL quadrants denote a high correlation between the occurrence of zoonotic outbreaks in the district and those in the neighboring district. In other words, if a district falls in HH category, then the neighboring districts also report a higher number of outbreaks. Similarly, LL indicates if there is a lower number of outbreaks in a district, then the neighboring districts also report a low numb of outbreaks. For both Moran's I and LISA values, a p-value of <0.05 was considered statistically significant. We used two layered India maps (state and district) to plot the distribution of outbreaks.^27^ Data analysis was conducted, and charts were visualized in R software.^28^

### Ethical considerations

We obtained reports from the publicly accessible IDSP weekly outbreak database. As the study utilized publicly available data, no ethical permission was required, and no personal or sensitive information was accessed, ensuring compliance with ethical standards.

### Role of the funding source

The present study is non-funded.

## Results

### Profile of zoonotic outbreaks

**Fig. 1:**
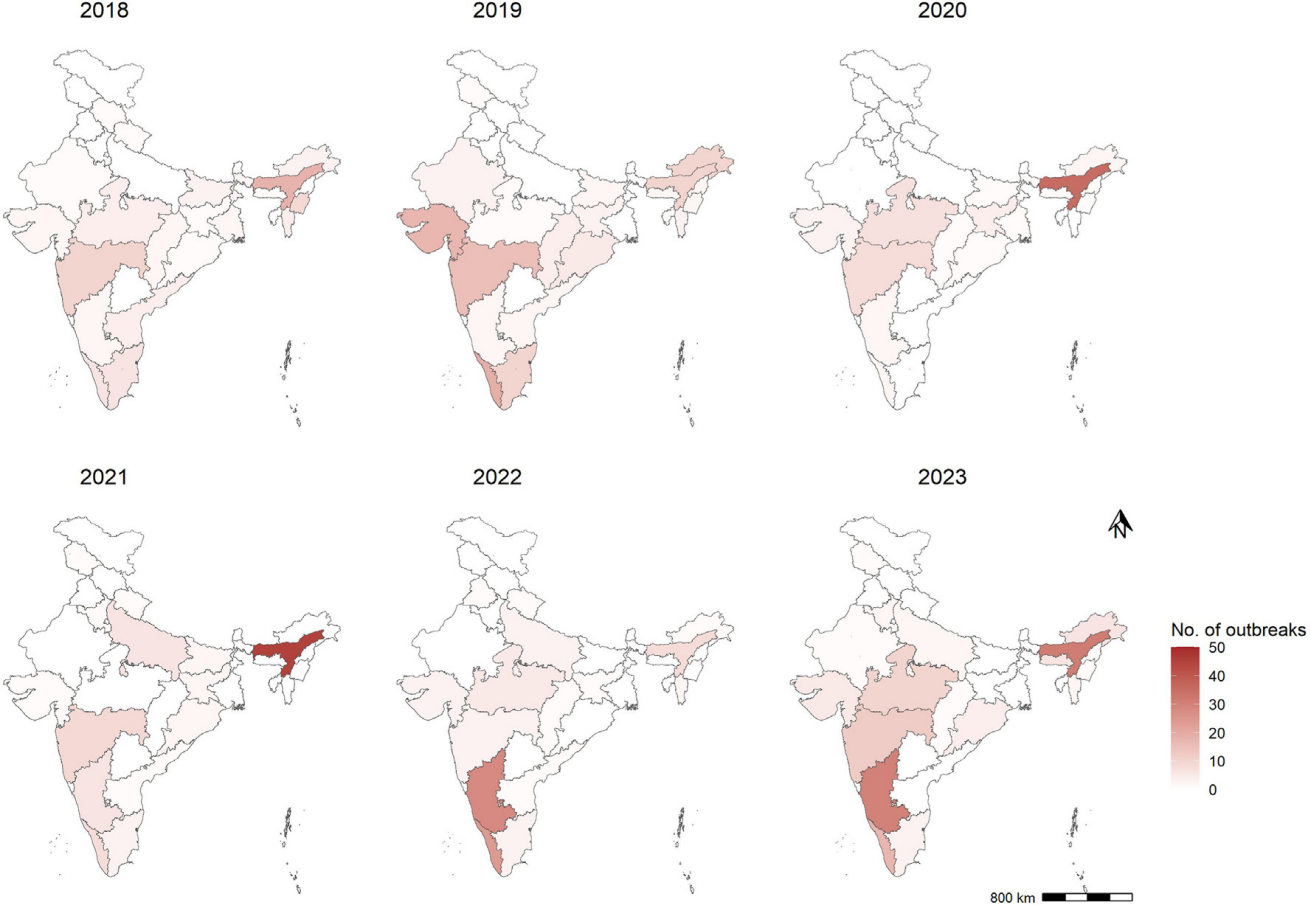
Distribution of zoonotic disease outbreaks reported under the Integrated Disease Surveillance Program, India by year and state, 2018–2023.

**Fig. 2:**
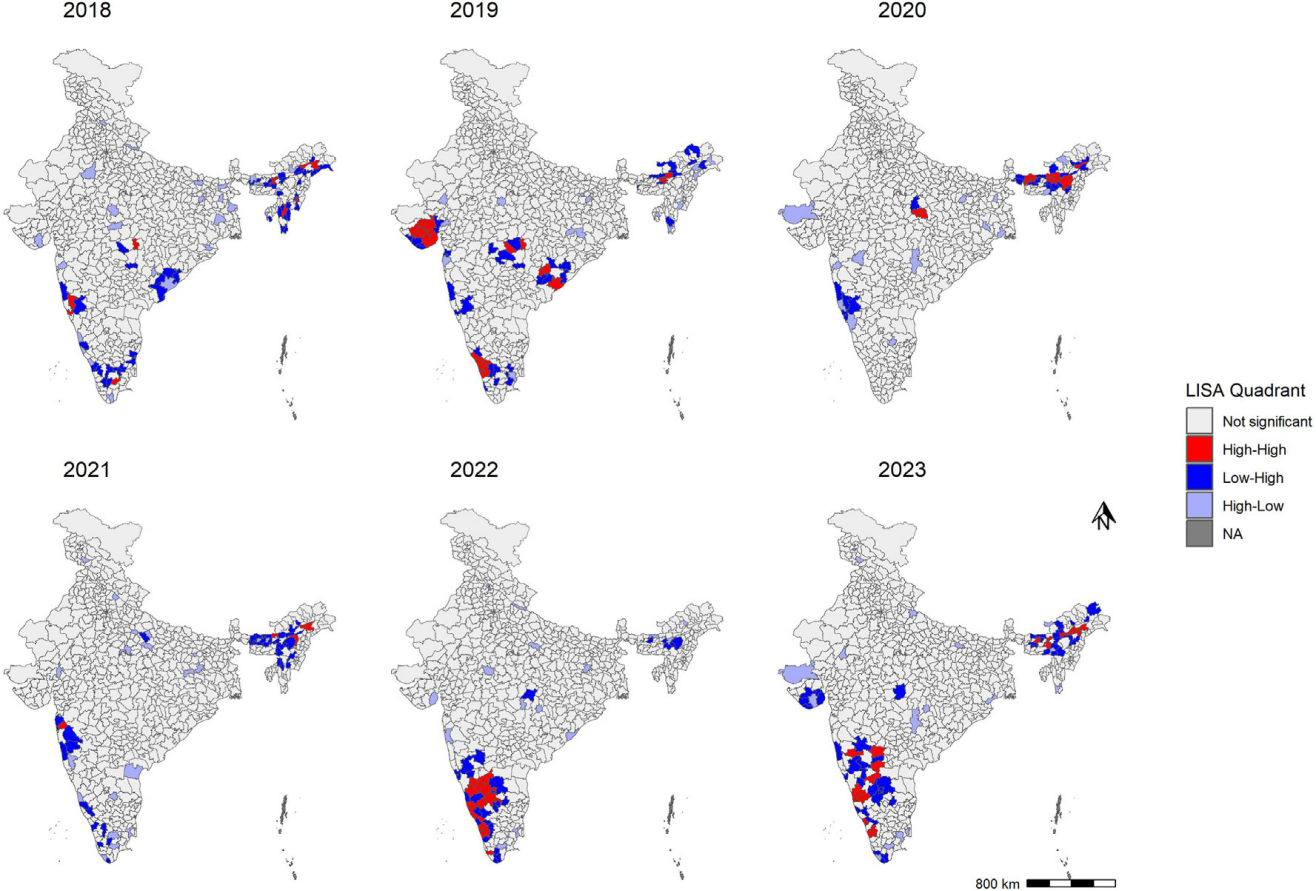
Spatial distribution of zoonotic disease outbreaks reported under the Integrated Disease Surveillance Program, India by year and district, 2018–2023.

Of the 6948 total outbreaks reported in the IDSP weekly outbreak system from 2018 to 2023, 583 (8.3%) were contributed by zoonotic diseases ranging 6–12%. Over the years, the number of zoonotic disease outbreaks increased significantly (unadjusted β: 0.11 (0.06–0.15), adjusted β: 0.07 (0.02–0.12)). The number of outbreaks increased by 58.8% in 2023 compared to 2022. The median number of zoonotic outbreaks reported per month was seven (IQR: 4–10). Zoonotic disease outbreaks consistently peaked during the months of June, July, and August across the study period. The majority of the zoonotic outbreaks were attributed to Japanese Encephalitis (29.5%, 172/583), followed by Leptospirosis (18.7%, 109/583), Scrub Typhus (13.9%, 81/583), and Rabies (10.6%, 81/583). The northeast region contributed 35.8% of zoonotic disease outbreaks, followed by the southern (31.7%) and western regions (15.4%) (Table 1). Assam state in the northeast region consistently documented higher reporting of zoonotic outbreaks. Karnataka and Kerala states, in the southern region of India, documented an increasing trend of zoonotic disease outbreaks between 2018 and 2023 (Fig. 1). The majority (94.2%) of the high-cluster districts in 2022–23 were concentrated in two regions of India (Southern (Karnataka and Kerala) and northeast (Assam)). In 2023, HH category districts include Ballari, Chikkamagaluru, Kalaburagi, Shivamogga, Udupi, and Raichur in Karnataka state, Kozhikode, Palakkad, and Thrissur in Kerala state, Ri-Bhoi in Meghalaya, and Sangli in Maharashtra. In Assam, multiple districts, including Barpeta, Biswanath, Darrang, Dibrugarh, Kamrup Metro, Lakhimpur, Majuli, and Sibsagar, were in the HH category (Fig. 2).

**Table 1:** Profile of zoonotic diseases reported by year and geographical region, India, 2018–2023.

Characteristics	2018			2019			2020			2021			2022			2023			Total	
	Number of outbreaks	Row %	Column %	Number of outbreaks	Row %	Column %	Number of outbreaks	Row %	Column %	Number of outbreaks	Row %	Column %	Number of outbreaks	Row %	Column %	Number of outbreaks	Row %	Column %	Number of outbreaks	Column %
**Disease**																				
Anthrax	5	31.3	5.7	7	43.8	6.0	1	6.3	1.4	1	6.3	1.1	0	0.0	0.0	2	12.5	1.5	16	2.7
Brucellosis	1	16.7	1.1	3	50.0	2.6	0	0.0	0.0	0	0.0	0.0	1	16.7	1.2	1	16.7	0.7	6	1.0
Crimean-Congo haemorrhagic fever	3	9.1	3.4	18	54.5	15.5	3	9.1	4.2	1	3.0	1.1	3	9.1	3.5	5	15.2	3.7	33	5.7
Hand foot mouth disease	2	28.6	2.3	1	14.3	0.9	0	0.0	0.0	1	14.3	1.1	3	42.9	3.5	0	0.0	0.0	7	1.2
Influenza	1	33.3	1.1	0	0.0	0.0	0	0.0	0.0	1	33.3	1.1	1	33.3	1.2	0	0.0	0.0	3	0.5
Japanese encephalitis	44	25.6	50.6	33	19.2	28.4	38	22.1	52.8	25	14.5	28.4	15	8.7	17.6	17	9.9	12.6	172	29.5
Kala azar	3	18.8	3.4	4	25.0	3.4	2	12.5	2.8	2	12.5	2.3	5	31.3	5.9	0	0.0	0.0	16	2.7
Kyasanur Forest disease	2	15.4	2.3	4	30.8	3.4	3	23.1	4.2	0	0.0	0.0	3	23.1	3.5	1	7.7	0.7	13	2.2
Leptospirosis	9	8.3	10.3	18	16.5	15.5	13	11.9	18.1	24	22.0	27.3	21	19.3	24.7	24	22.0	17.8	109	18.7
Leptospirosis & scrub typhus	0	0.0	0.0	1	33.3	0.9	1	33.3	1.4	1	33.3	1.1	0	0.0	0.0	0	0.0	0.0	3	0.5
Melioidosis	3	60.0	3.4	1	20.0	0.9	0	0.0	0.0	1	20.0	1.1	0	0.0	0.0	0	0.0	0.0	5	0.9
Monkey pox	0	0.0	0.0	0	0.0	0.0	0	0.0	0.0	0	0.0	0.0	6	54.5	7.1	5	45.5	3.7	11	1.9
Nipah	1	50.0	1.1	1	50.0	0.9	0	0.0	0.0	0	0.0	0.0	0	0.0	0.0	0	0.0	0.0	2	0.3
Noro virus	0	0.0	0.0	0	0.0	0.0	0	0.0	0.0	0	0.0	0.0	4	100.0	4.7	0	0.0	0.0	4	0.7
Rabies	0	0.0	0.0	0	0.0	0.0	0	0.0	0.0	9	14.5	10.2	9	14.5	10.6	44	71.0	32.6	62	10.6
Scrub typhus	10	12.3	11.5	14	17.3	12.1	11	13.6	15.3	15	18.5	17.0	10	12.3	11.8	21	25.9	15.6	81	13.9
Snake bite	0	0.0	0.0	0	0.0	0.0	0	0.0	0.0	0	0.0	0.0	0	0.0	0.0	1	100.0	0.7	1	0.2
Trypanosomiasis	0	0.0	0.0	1	100.0	0.9	0	0.0	0.0	0	0.0	0.0	0	0.0	0.0	0	0.0	0.0	1	0.2
West Nile fever	0	0.0	0.0	10	58.8	8.6	0	0.0	0.0	0	0.0	0.0	2	11.8	2.4	5	29.4	3.7	17	2.9
Zika virus	3	14.3	3.4	0	0.0	0.0	0	0.0	0.0	7	33.3	8.0	2	9.5	2.4	9	42.9	6.7	21	3.6
**Region**																				
Central	6	12.8	6.9	5	10.6	4.3	8	17.0	11.1	6	12.8	6.8	9	19.1	10.6	13	27.7	9.6	47	8.1
Eastern	7	20.0	8.0	10	28.6	8.6	7	20.0	9.7	6	17.1	6.8	1	2.9	1.2	4	11.4	3.0	35	6.0
Northeast	36	17.2	41.4	30	14.4	25.9	40	19.1	55.6	45	21.5	51.1	11	5.3	12.9	47	22.5	34.8	209	35.8
Northern	3	17.6	3.4	5	29.4	4.3	0	0.0	0.0	3	17.6	3.4	2	11.8	2.4	4	23.5	3.0	17	2.9
Southern	22	11.9	25.3	33	17.8	28.4	6	3.2	8.3	18	9.7	20.5	56	30.3	65.9	50	27.0	37.0	185	31.7
Western	13	14.4	14.9	33	36.7	28.4	11	12.2	15.3	10	11.1	11.4	6	6.7	7.1	17	18.9	12.6	90	15.4
**Total**	**87**	**14.9**	**100.0**	**116**	**19.9**	**100.0**	**72**	**12.3**	**100.0**	**88**	**15.1**	**100.0**	**85**	**14.6**	**100.0**	**135**	**23.2**	**100.0**	**583**	**100.0**

Of the 583 reported outbreaks, 203 (34.8%) were reported in the pre-COVID period, 160 (27.4%) in the COVID period, and 220 (37.7%) in the post-COVID period. We observed a decline in the number of outbreaks during the COVID period compared to the pre-COVID period (β = −0.18, p = 0.10). There was a significant increase in the number of outbreaks in the post-COVID period (β = 0.33, p = 0.002) compared to the COVID period. Although the number of outbreaks increased in the post-COVID period (β = 0.15, p = 0.14) compared to the pre-COVID period, the change was not statistically significant.

### Case fatality ratio per 100 cases

Out of 583 zoonotic outbreaks reported by IDSP, 90.9% of outbreaks had information on the number of cases and deaths. Of the 53 outbreaks that did not document the number of cases and deaths, 77.3% belong to the reporting year 2018. Over the years, the proportion of outbreaks with missing values of the number of cases and deaths declined. The average number of cases reported per outbreak was 36 (Median: 17; IQR: 9–36). Overall, CFR was 6.9% (294/4236). CFR was maximum in the western region (12.7%, 64/502) (Table 2, Table 3).

**Table 2:** Distribution of case fatality ratio (per 100 cases) and zoonotic outbreaks (OB) reported late by year and geographical region, India, 2018–23.

CFR per 100 cases																					
Year	Central			Eastern			Northeast			Northern			Southern			Western			Overall		
	No. cases	No. deaths	CFR	No. cases	No. deaths	CFR	No. cases	No. deaths	CFR	No. cases	No. deaths	CFR	No. cases	No. deaths	CFR	No. cases	No. deaths	CFR	No. cases	No. deaths	CFR
2018	265	6	2.3	67	1	1.5	579	8	1.4	23	0	0.0	385	8	2.1	16	1	6.3	1335	24	1.8
2019	14	4	28.6	90	3	3.3	451	19	4.2	46	0	0.0	235	13	5.5	217	41	18.9	1053	80	7.6
2020	33	3	9.1	54	2	3.7	191	22	11.5	0	0	0.0	8	1	12.5	31	9	29.0	317	37	11.7
2021	24	1	4.2	36	2	5.6	65	19	29.2	9	8	88.9	181	8	4.4	88	4	4.5	403	42	10.4
2022	43	2	4.7	1	0	0.0	21	5	23.8	10	0	0.0	200	24	12.0	58	2	3.4	333	33	9.9
2023	129	2	1.6	32	1	3.1	358	36	10.1	45	5	11.1	139	27	19.4	92	7	7.6	795	78	9.8
Total	508	18	3.5	280	9	3.2	1665	109	6.5	133	13	9.8	1148	81	7.1	502	64	12.7	4236	294	6.9

Reported late																					
Year	Central			Eastern			Northeast			Northern			Southern			Western			Overall		
	No. OB	No. OB reported late	%	No. OB	No. OB reported late	%	No. OB	No. OB reported late	%	No. OB	No. OB reported late	%	No. OB	No. OB reported late	%	No. OB	No. OB reported late	%	No. OB	No. OB reported late	%
2018	6	2	33.3	7	3	42.9	36	22	61.1	3	0	0.0	22	7	31.8	13	9	69.2	87	43	49.4
2019	5	1	20.0	10	5	50.0	30	16	53.3	5	2	40.0	33	16	48.5	33	21	63.6	116	61	52.6
2020	8	0	0.0	7	2	28.6	40	16	40.0	0	0	0.0	6	1	16.7	11	8	72.7	72	27	37.5
2021	6	2	33.3	6	1	16.7	45	19	42.2	3	1	33.3	18	8	44.4	10	5	50.0	88	36	40.9
2022	9	3	33.3	1	0	0.0	11	8	72.7	2	1	50.0	56	13	23.2	6	3	50.0	85	28	32.9
2023	13	3	23.1	4	0	0.0	47	0	0.0	4	0	0.0	50	2	4.0	17	2	11.8	135	7	5.2
Total	47	11	23.4	35	11	31.4	209	81	38.8	17	4	23.5	185	47	25.4	90	48	53.3	583	202	34.6

**Table 3:** Distribution of case fatality ratio (per 100 cases) and zoonotic outbreaks reported late by type of disease, India, 2018–23.

Disease	No. zoonotic outbreaks (a)	No. outbreaks reported late (b)	% delayed (b/a∗100)^a^	Total no. of Cases (c)	No. of deaths (d)	Case fatality rate per 100 cases (d/c∗100)^a^
Anthrax	16	3	18.8	103	1	1.0
Brucellosis	6	4	66.7	22	0	0.0
Crimean-Congo haemorrhagic fever	33	16	48.5	39	17	43.6
Hand foot mouth disease	7	1	14.3	127	0	0.0
Influenza	3	1	33.3	60	1	1.7
Japanese encephalitis	172	76	44.2	737	83	11.3
Kala Azar	16	7	43.8	19	2	10.5
Kyasanur forest disease	13	6	46.2	30	11	36.7
Leptospirosis	109	38	34.9	905	61	6.7
Leptospirosis & scrub typhus	3	1	33.3	11	1	9.1
Melioidosis	5	3	60.0	4	2	50.0
Monkey pox	11	0	0.0	14	1	7.1
Nipah	2	1	50.0	10	7	70.0
Noro virus^a^	4	1	25.0	NA	NA	NA
Rabies	62	6	9.7	75	66	88.0
Scrub typhus	81	25	30.9	1683	35	2.1
Snake bite	1	0	0.0	1	1	100.0
Trypanosomiasis	1	1	100.0	1	0	0.0
West Nile fever	17	10	58.8	20	1	5.0
Zika virus	21	2	9.5	375	4	1.1
**Total**	583	202	34.6	4236	294	6.9

a Number of cases and deaths not reported in IDSP database for Norovirus outbreak.

### Zoonotic outbreaks reported late

Of 583 zoonotic disease outbreaks reported, 34.6% were reported late, and 2.8% had follow-up reports on the investigation. We observed a decline in the proportion of outbreaks reported late among the total zoonotic outbreaks reported over the years [52.6% (61/116) in 2019, 40.9% (36/88) in 2021, and 5.2% (7/135) in 2023] (Table 2 and Supplementary Table S2). Over 50% of the outbreaks were reported late for diseases such as West Nile fever (59%, 10/17) and Brucellosis (66.7%, 4/6). Over half of the outbreaks were reported late by the Western region (53.3%, 48/90) (Table 2). Of the 202 reported late zoonotic outbreaks, 29.7% were contributed by Assam state of the northeast region. Zoonotic disease outbreaks reported late by Assam declined over the years from 2018 to 2023 [70% (7/10) in 2019, 42.2% (19/45) in 2021, and 0% (0/31) in 2023]. Similarly, Gujarat and Karnataka showed a declining trend in reporting outbreaks late (Supplementary Table S3). Of the 4236 cases reported, 25.2% were contributed by the outbreaks reported late. CFR among the outbreaks reported late was 9.4% (101/1070).

### Pattern of key zoonotic disease outbreaks

#### Japanese encephalitis

**Fig. 3:**
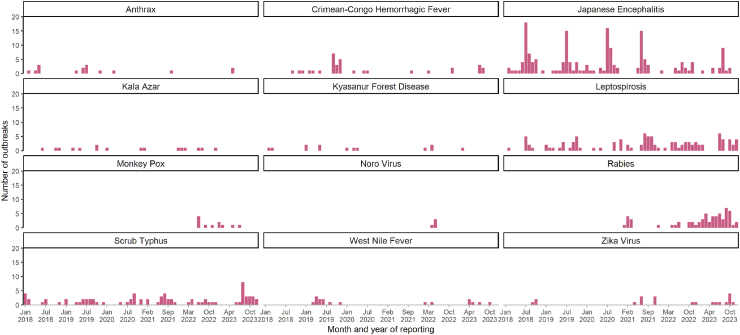
Distribution of priority zoonotic disease outbreaks by month, India, 2018–23.

Over the years, there has been a decline in the JE outbreaks reported from 2018 to 2023. However, the reporting consistently peaked in the months of June–August (Fig. 3). Of the total 172 JE outbreaks, 66.9% were reported in the Northeast region (Supplementary Table S4). Over one-third (44.2%, 76/172) of the JE outbreaks were reported late between 2018 and 2023 (Table 3). However, there has been a decline in the proportion of late reporting of JE outbreaks over the years (Supplementary Table S2). The Western (66.7%, 10/15) and Northeast region (47%, 54/115) reported a higher proportion of JE outbreaks reported late (Supplementary Table S5).

#### Leptospirosis

We documented an increasing trend of leptospirosis reporting in the IDSP. Of the 109 leptospirosis outbreaks reported, 45% were reported by the southern region (Supplementary Table S4). The eastern region has not reported any leptospirosis outbreaks. In the southern region, leptospirosis was reported by the states of Karnataka (42.8%, 21/49), Tamilnadu (40.8%, 20/49), and Kerala (16.3%, 8/49). In the northeast region, Assam state reported leptospirosis (95.6%, 22/23). In the western region, the majority of leptospirosis outbreaks were reported by Maharashtra state (95.4%, 21/22). One-third of the leptospirosis outbreaks were reported late (34.8%). Over 10% of the leptospirosis outbreaks were reported late consistently over the years (Supplementary Table S2). The western region reported a higher proportion of leptospirosis outbreaks reported late (63.6%, 14/22) followed by the northeast region (47.8%, 11/23) (Supplementary Table S5).

#### Scrub typhus

Similar to leptospirosis, there was an increasing trend in scrub typhus outbreak reporting between 2018 and 2023 (Fig. 3). Of the 81 scrub typhus outbreaks, 48.1% were reported by the northeastern region, followed by the central region (18.5%) (Supplementary Table S4). Assam, Mizoram, and Maharashtra states contributed to the majority of scrub typhus outbreaks. Around one-third of the scrub typhus outbreaks were reported late (30.9%, 25/81).

#### Rabies

We observed that rabies outbreaks increased steeply in 2023 compared to 2022 [14.5% (9/62) in 2022 to 34.8% (44/62) in 2023]. The southern region (54.8%, 34/62) and the northeast region (43.5%, 27/62) contributed to the majority of rabies outbreaks (Supplementary Table S4). Karnataka state of the southern region accounted for 53.2% (33/62) of the rabies outbreaks. One in ten rabies outbreaks were reported late (9.7%, 6/62) with a CFR of 88% (66/75).

#### Other diseases

We documented that all 31 zoonotic outbreaks reported by Gujarat were CCHF. Of them, 51.6% (16/31) were reported late. CFR of CCHF was over 30% in all the reported years. Karnataka state reported around one-third of the KFD outbreaks in India (38.4%, 5/13). Two-thirds of kala-azar outbreaks were reported in Kerala state (68.7%, 11/16), and 56.2% (9/16) of Anthrax outbreaks were reported in Odisha state.

### Emerging and re-emerging outbreaks

In 2023, we documented Maharashtra's first recorded outbreak of the Zika virus disease. Additionally, we observed the re-emergence of West Nile Fever in the Kerala state of southern region in 2022 after a three-year gap. Monkeypox and norovirus disease outbreaks were also recorded for the first time in Kerala in 2022.

## Discussion

Our analysis provides a comprehensive, national-level overview of zoonotic disease outbreaks, offering insights into emerging patterns and trends across the country. One in ten outbreaks notified by IDSP was of zoonotic origin. We documented an increase in the number of zoonotic disease outbreaks in 2023.

The distribution of infectious disease outbreaks in India exhibits regional variations. Three-fourths of the zoonotic outbreaks were reported in the northeast region. In the northeastern region, JE, rabies, and scrub typhus were the commonly reported outbreaks. The eastern region reported fewer outbreaks compared to the other regions. However, JE accounted for around half of the reported outbreaks in the eastern region, with predominant reporting from the Jharkhand state. Around two-thirds of the outbreaks reported in the central region were leptospirosis and scrub typhus. In the western region, all the CCHF outbreaks are reported by the Gujarat state, and leptospirosis is focused on the state of Maharashtra, India. Half of the outbreaks in the southern region were accounted for by leptospirosis and rabies, specifically in the coastal states of India, including Karnataka and Tamilnadu. This is consistent with existing evidence on reporting of outbreaks from respective geographical regions.^22^ We documented the outbreak clustering in districts of the southern region states of Karnataka and Kerala, along with Assam from the northeastern region, during 2022 and 2023. Prioritizing diseases at the state and regional levels based on outbreak patterns is critical for designing effective public health interventions, allocating resources efficiently, and strengthening the overall healthcare system. Additionally, building the capacity of epidemiologists in disease-specific outbreak investigation and case management is vital. States in the respective region can consider adopting the One Health approach by collaborating with the regional and national institutes for disease-specific surveillance system strengthening.

In our analysis, a single outbreak of trypanosomiasis was reported in Delhi, India. However, active transmission of the disease has not been documented in the country, suggesting that it is likely due to imported cases or arising from sporadic infections, with minimal or no potential for further spread. Given the absence of endemic transmission, this event may reflect an isolated occurrence rather than a true outbreak. Further epidemiological investigation would be necessary to determine the source of infection and assess the risk of local transmission. Strengthening surveillance and laboratory confirmation mechanisms can help in accurately identifying and classifying such cases in the future.

In our analysis, JE, leptospirosis, and scrub typhus outbreaks followed a seasonal pattern with increased reporting during the June–September months (monsoon season) of each year. Studies documented that the human JE cases peaked during the monsoon and post-monsoon periods, correlating with the notification of outbreaks observed in our analysis.^29^ The seasonal pattern of JE correlates with the findings of the high seropositivity of the pig population (22.9%) during the winter season in various states of India, including Assam.^30^^,^^31^ The incidence of JE cases might be due to the high mosquito index and seroconversion in pigs during pre-monsoon and monsoon seasons.^30^ The burden of JE is predominantly concentrated in Southeast Asia, with India and China accounting for over 90% of reported cases. India has documented a fatality burden more than five times higher than that of China.^32^ Similar to JE, coastal regions in India reported higher incidence of leptospirosis outbreaks in the monsoon season due to frequent flooding from the rivers, aggravating the transmission of Leptospira from the contaminated waters with urine of rats and rodents.^33^^,^^34^ In Tamilnadu state, India, seroprevalence of leptospirosis among rats and cattle was observed as 51% and 87%.^35^ In a meta-analysis of serosurvey results across India, the prevalence was found to be 40% in rodents, 24% in cattle, and 15% in pigs.^36^ With rapid urbanization, inadequate sanitation infrastructure with the accumulation of stagnant water and waste further creates breeding grounds for rodents that carry Leptospira.^37^ Around half of the Scrub typhus outbreaks were reported in the Northeast region with increased incidence during the rainy and post-monsoon season. Studies in the northeastern states of India indicate that chigger mite infestation rates are highest during and after the monsoon season, correlating with increased humidity and temperature. Findings revealed a high chigger infestation rate, with Meghalaya and Mizoram showing infestation indices exceeding the critical threshold of 0.69 per rodent (Meghalaya: 1.80, Mizoram: 12.3–19.2).^38^, ^39^, ^40^ The studies indicate the presence of vector mites was higher in this region, correlating with the findings of increased incidence of scrub typhus outbreaks in our analysis. Environmental factors such as monsoon rains, vegetation density, and rodent populations influence vector abundance, leading to heightened transmission risk in the post-monsoon months. Strengthening surveillance efforts during pre-monsoon and monsoon months, particularly in areas with high animal seropositivity, would be beneficial in anticipating and mitigating higher transmission levels.

We documented a decline in the reporting of zoonotic disease outbreaks during the COVID-19 pandemic, followed by an increase in the number of outbreaks during 2022–23. The finding is similar to the infectious disease surveillance of countries like Japan, Russia, and England, where there was a decline in reporting during the pandemic.^41^, ^42^, ^43^ In Russia, infectious disease notification declined by over 60% during 2020–21.^42^ Similarly, there was a 52% reduction in gastrointestinal outbreaks compared to the pre-COVID period in England, United Kingdom.^43^ In Germany, the surveillance system detected 35% lower cases of infectious diseases in 2020.^44^ The decline of outbreak notification of routine surveillance diseases during the pandemic might be due to lower prioritization of testing and diagnosis, limited healthcare professionals, and reduced motivation for reporting due to a lack of feedback.^45^ However, the continuous analysis of disease outbreaks will aid in understanding the precise estimate and allocation of resources in the future.

Over one-tenth of the leptospirosis and scrub typhus disease outbreaks were consistently reported late between 2018 and 2023. Around half of the CCHF outbreaks were reported late. The western region of India reported around half of the outbreaks late, with the states of Maharashtra and Gujarat being predominant. However, over the years, the proportion of outbreaks reported late declined in all geographical regions of India. The reduction in delayed outbreak reporting is likely attributed to the implementation of IHIP in 2021, where event alerts are generated in real-time and promptly notified at the state and national levels.^46^ However, high CFR diseases such as CCHF, JE, and KFD were reported to be delayed by more than 40%. This delay in notification could result in postponed outbreak investigations, potentially leading to inadequate public health responses. Therefore, evaluating the outbreak surveillance systems could provide critical reasons for delayed reporting, enabling the stakeholders to improve the timeliness of reporting and undertake public health actions.

Maharashtra, Karnataka, Kerala, and Assam states of India have consistently reported zoonotic outbreaks during 2018–23. Kerala reported emerging and re-emerging diseases, including Zika, Norovirus, and West Nile fever. The reason for the increased reporting of zoonotic outbreaks in Kerala could be the state's dependence on animal protein, which has been linked to the reemergence of foodborne zoonoses, including those arising from improper food handling and preparation.^47^ We documented an increasing trend in leptospirosis and scrub typhus outbreaks. Agricultural practices involving close interaction with livestock contribute to outbreaks such as leptospirosis, particularly among workers who handle animals or animal products.^47^ Additionally, the disruption of ecosystems through deforestation or the encroachment of human activities into wild habitats, including agroforestry, can catalyze the emergence or transmission of diseases to human populations. Migratory birds, which frequent Kerala's water bodies, contribute to the spread of avian diseases like the West Nile virus.^48^ Other factors contributing to the reporting of zoonotic outbreaks in India could include enhanced surveillance capacity to detect and confirm outbreaks. In states where outbreaks are consistently identified, the surveillance systems are likely robust and effective.

In our analysis from the national outbreak surveillance system, there could be a possibility of reporting bias (underreporting and overreporting) of key indicators. Not all the outbreaks from the community level may be captured by the system and reported in the outbreak database due to differences in surveillance sensitivity across regions, limited health system capacity, or variations in reporting practices. Conversely, certain outbreaks might be overreported due to heightened surveillance in certain regions due to the availability of a higher proportion of trained human resources in comparison to other regions. Also, since the analysis relied on notified outbreaks reported to the IDSP, there may be a potential underestimation in the characterization of outbreaks that was missed by the system. Hence, we recommend in the future to triangulate the IDSP outbreak data with other sources, including state health reports for comprehensive assessment. In addition, we recommend evaluating the outbreak surveillance system to assess the sensitivity of the surveillance system.

Secondly, the IDSP weekly outbreak dashboard captures information on the outbreaks, including the number of cases and deaths at the time of earliest point of notification. However, there is a likelihood that laboratory confirmation, case classification, and revisions in case and death counts may occur later in the outbreak, following the deployment of the Rapid Response Team and a systematic outbreak investigation. In addition, the number of cases and deaths used for estimating the CFR in our analysis could be under or overestimated as the number of cases and deaths at the end of the outbreak investigation (two incubation periods from the last case reported) might vary, which could have led to potential bias in inferring CFR. However, with the implementation of IHIP since 2021, real-time updates on the number of cases and deaths, laboratory information, trawling information through RRT, and case definition used will be accessible. To mitigate the limitations of under- or over-estimation of CFR and outbreak burden, we recommend integrating IHIP data into the weekly outbreak reporting dashboard. This will help estimate the number of event alerts generated, number confirmed as outbreaks and investigated, enabling more accurate and precise trend analysis. This information will also help determine the percentage of lab-confirmed outbreaks, facilitating the identification of gaps, and strengthening the laboratory information system for improved outbreak reporting in the future. Furthermore, the total cases and deaths reported at the end of the outbreak will aid in the estimation of the outbreak and event alert threshold for priority diseases at the individual state-level.

Thirdly, some districts in India might have better reporting mechanisms than others, leading to a concentration of outbreaks in certain regions. To address this special bias, we analyzed the outbreaks trend over years concretely by adjusting the districts and inferred using spatial clustering for change in trend over years. However, future studies could evaluate the reporting capability of the system by assessing the trained human power and other health system pillars and estimate the population-adjusted outbreak rates to standardize comparisons. The weekly outbreak reports lack key information, including the date of outbreak onset and the date of reporting for delayed outbreaks, which prevented us from calculating the median reporting delay. Finally, in the context of no outbreaks flagged in a particular state in a particular reporting week, the state was expected to report “NIL” to the IDSP central unit. Later, IDSP compiles and displays the indicator named “number of states submitted outbreaks including NIL reports” in the weekly report. This indicator documents the reporting quality and completeness of the reporting from the states. However, we observed that the indicator is not displayed in all the reporting weeks of the analysis period (2018–23). Strengthening “NIL” reporting is essential to maintain the accuracy and effectiveness of India's disease surveillance system. The missing indicator could be due to the irregular submission of NIL reports by states or technical or system-related issues affecting report generation. To ensure consistent documentation of reporting quality, IDSP shall standardize the inclusion of this indicator in all weekly reports, regardless of state-level reporting compliance. Since the present analysis is based on the data reported to the IDSP, the generalizability of these findings is limited to those outbreaks captured by the weekly reports.

We recommend incorporating the operational definitions for delayed and follow-up outbreaks, along with the inclusion of the outbreak onset date and reporting date, to estimate delays in days. We recommend estimating the alert, outbreak thresholds and specific delay cut-off for each reporting diseases at individual reporting unit or community levels, which may guide the field workers to identify and undertake the actions timely. Integrating human disease surveillance with veterinary surveillance, along with strengthening laboratory capacity, can significantly enhance the detection, monitoring, and response to zoonotic diseases. A collaborative One Health approach will allow for the early identification of emerging zoonotic threats by capturing data on animal health trends, potential spillover events, and environmental risk factors. Additionally, cross-sectoral data sharing will improve outbreak investigations, leading to more timely and evidence-based public health interventions.^49^^,^^50^ We recommend establishing a structured mechanism for real-time data exchange between human and animal health sectors, alongside capacity-building initiatives to improve diagnostic accuracy and outbreak response.

We documented the trends and patterns of India's national-level outbreak surveillance system and mapped the priority zoonotic diseases by geographical region. We outlined the limitations in the documentation process and reporting format of the weekly outbreak surveillance report. We recommend for robust documentation practices, enhanced training for field teams, and integration of laboratory confirmation data for better outbreak characterization, that will facilitate a better understanding of the epidemiology of zoonotic infectious diseases, enabling the formulation of targeted recommendations at regional-level.

## Contributors

Conceptualization: MK, MR, MS, AR; Data curation: MK, MR; Data cleaning and Formal analysis: MK, MR, MS, AR; Accessed and verified data: MK, MR, MS; Methodology: MK, MR, MS, AR; Validation: MK, MR, MS, AR; Visualization: MK, MR, MS; Writing – original draft: MK, MR, MS, AR; Responsibility for the decision to submit the manuscript: MK, MR, AR.

## Data sharing statement

Data will be shared upon reasonable request to the corresponding author.

## Editor note

The Lancet Group takes a neutral position with respect to territorial claims in published maps and institutional affiliations.

## Declaration of generative AI and AI-assisted technologies in the writing process

Authors have not used Artificial Intelligence in drafting the manuscript.

## Declaration of interests

The authors declare that they have no competing interests.
