# Supplementary material for: Zoonotic disease outbreaks reported under India's Integrated Disease Surveillance Programme, 2018–2023: a cross-sectional analysis of national surveillance data
Source: Lancet Reg Health Southeast Asia. 2025 May 20;37:100601. doi: 10.1016/j.lansea.2025.100601 (PMC12148586; doi:10.1016/j.lansea.2025.100601)
Supplement: Supplementary Tables S1–S5 [file mmc1.docx]

**Supplementary table 1: Distribution of states by divisions, India**

| **State** | **Division** |
| --- | --- |
| Chhattisgarh | Central |
| Madhya Pradesh | Central |
| Uttar Pradesh | Central |
| Bihar | Eastern |
| Jharkhand | Eastern |
| Odisha | Eastern |
| West Bengal | Eastern |
| Arunachal Pradesh | Northeast |
| Assam | Northeast |
| Manipur | Northeast |
| Meghalaya | Northeast |
| Mizoram | Northeast |
| Nagaland | Northeast |
| Sikkim | Northeast |
| Tripura | Northeast |
| Chandigarh | Northern |
| Delhi | Northern |
| Haryana | Northern |
| Himachal Pradesh | Northern |
| Jammu and Kashmir | Northern |
| Ladakh | Northern |
| Punjab | Northern |
| Rajasthan | Northern |
| Uttarakhand | Northern |
| A&N Islands | Southern |
| Andhra Pradesh | Southern |
| Karnataka | Southern |
| Kerala | Southern |
| Lakshadweep | Southern |
| Puducherry | Southern |
| Tamil Nadu | Southern |
| Telangana | Southern |
| Dadar and Nagar Haveli | Western |
| Daman and Diu | Western |
| Goa | Western |
| Gujarat | Western |
| Maharashtra | Western |

**Supplementary table 2: Distribution of zoonotic disease outbreaks (OB) reported late by disease type and year, India, 2018-23**

| **Disease** | **2019** | | | **2019** | | | **2020** | | | **2021** | | | **2022** | | | **2023** | | |
| --- | --- | --- | --- | --- | --- | --- | --- | --- | --- | --- | --- | --- | --- | --- | --- | --- | --- | --- |
|  | **No. OB** | **No. OB Reported late** | **%** | **No. OB** | **No. OB Reported late** | **%** | **No. OB** | **No. OB Reported late** | **%** | **No. OB** | **No. OB Reported late** | **%** | **No. OB** | **No. OB Reported late** | **%** | **No. OB** | **No. OB Reported late** | **%** |
| Anthrax | 5 | 0 | 0.0 | 7 | 3 | 42.9 | 1 | 0 | 0.0 | 1 | 0 | 0.0 | 0 | 0 | 0.0 | 2 | 0 | 0.0 |
| Brucellosis | 1 | 1 | 100.0 | 3 | 2 | 66.7 | 0 | 0 | 0.0 | 0 | 0 | 0.0 | 1 | 1 | 100.0 | 1 | 0 | 0.0 |
| Crimean-Congo Haemorrhagic Fever | 3 | 0 | 0.0 | 18 | 11 | 61.1 | 3 | 2 | 66.7 | 1 | 1 | 100.0 | 3 | 2 | 66.7 | 5 | 0 | 0.0 |
| Hand Foot Mouth Disease | 2 | 0 | 0.0 | 1 | 0 | 0.0 | 0 | 0 | 0.0 | 1 | 0 | 0.0 | 3 | 1 | 33.3 | 0 | 0 | 0.0 |
| Influenza | 1 | 0 | 0.0 | 0 | 0 | 0.0 | 0 | 0 | 0.0 | 1 | 1 | 100.0 | 1 | 0 | 0.0 | 0 | 0 | 0.0 |
| Japanese Encephalitis | 44 | 27 | 61.4 | 33 | 16 | 48.5 | 38 | 16 | 42.1 | 25 | 11 | 44.0 | 15 | 6 | 40.0 | 17 | 0 | 0.0 |
| Kala Azar | 3 | 2 | 66.7 | 4 | 2 | 50.0 | 2 | 0 | 0.0 | 2 | 2 | 100.0 | 5 | 1 | 20.0 | 0 | 0 | 0.0 |
| Kyasanur Forest Disease | 2 | 2 | 100.0 | 4 | 2 | 50.0 | 3 | 0 | 0.0 | 0 | 0 | 0.0 | 3 | 2 | 66.7 | 1 | 0 | 0.0 |
| Leptospirosis | 9 | 4 | 44.4 | 18 | 7 | 38.9 | 13 | 7 | 53.8 | 24 | 9 | 37.5 | 21 | 7 | 33.3 | 24 | 4 | 16.7 |
| Leptospirosis & Scrub typhus | 0 | 0 | 0.0 | 1 | 0 | 0.0 | 1 | 0 | 0.0 | 1 | 1 | 100.0 | 0 | 0 | 0.0 | 0 | 0 | 0.0 |
| Melioidosis | 3 | 2 | 66.7 | 1 | 0 | 0.0 | 0 | 0 | 0.0 | 1 | 1 | 100.0 | 0 | 0 | 0.0 | 0 | 0 | 0.0 |
| Monkey Pox | 0 | 0 | 0.0 | 0 | 0 | 0.0 | 0 | 0 | 0.0 | 0 | 0 | 0.0 | 6 | 0 | 0.0 | 5 | 0 | 0.0 |
| Nipah | 1 | 0 | 0.0 | 1 | 1 | 100.0 | 0 | 0 | 0.0 | 0 | 0 | 0.0 | 0 | 0 | 0.0 | 0 | 0 | 0.0 |
| Noro Virus | 0 | 0 | 0.0 | 0 | 0 | 0.0 | 0 | 0 | 0.0 | 0 | 0 | 0.0 | 4 | 1 | 25.0 | 0 | 0 | 0.0 |
| Rabies | 0 | 0 | 0.0 | 0 | 0 | 0.0 | 0 | 0 | 0.0 | 9 | 2 | 22.2 | 9 | 3 | 33.3 | 44 | 1 | 2.3 |
| Scrub Typhus | 10 | 5 | 50.0 | 14 | 8 | 57.1 | 11 | 2 | 18.2 | 15 | 6 | 40.0 | 10 | 2 | 20.0 | 21 | 2 | 9.5 |
| Snake bite | 0 | 0 | 0.0 | 0 | 0 | 0.0 | 0 | 0 | 0.0 | 0 | 0 | 0.0 | 0 | 0 | 0.0 | 1 | 0 | 0.0 |
| Trypanosomiasis | 0 | 0 | 0.0 | 1 | 1 | 100.0 | 0 | 0 | 0.0 | 0 | 0 | 0.0 | 0 | 0 | 0.0 | 0 | 0 | 0.0 |
| West Nile Fever | 0 | 0 | 0.0 | 10 | 8 | 80.0 | 0 | 0 | 0.0 | 0 | 0 | 0.0 | 2 | 2 | 100.0 | 5 | 0 | 0.0 |
| Zika Virus | 3 | 0 | 0.0 | 0 | 0 | 0.0 | 0 | 0 | 0.0 | 7 | 2 | 28.6 | 2 | 0 | 0.0 | 9 | 0 | 0.0 |
| **Total** | **87** | **43** | 49.4 | **116** | **61** | 52.6 | **72** | **27** | 37.5 | **88** | **36** | 40.9 | **85** | **28** | 32.9 | **135** | **7** | 5.2 |

**Supplementary table 3: Distribution of zoonotic disease outbreaks (OB) reported late by disease type and states, India, 2018-23**

| **State** | **2018** | | | **2019** | | | **2020** | | | **2021** | | | **2022** | | | **2023** | | | **Overall** | | |
| --- | --- | --- | --- | --- | --- | --- | --- | --- | --- | --- | --- | --- | --- | --- | --- | --- | --- | --- | --- | --- | --- |
|  | **No. OB** | **No. OB Reported late** | **%** | **No. OB** | **No. OB Reported late** | **%** | **No. OB** | **No. OB Reported late** | **%** | **No. OB** | **No. OB Reported late** | **%** | **No. OB** | **No. OB Reported late** | **%** | **No. OB** | **No. OB Reported late** | **%** | **No. OB** | **No. OB Reported late** | **%** |
| Andhra Pradesh | 4 | 0 | 0.0 | 2 | 1 | 50.0 | 0 | 0 | 0.0 | 1 | 0 | 0.0 | 1 | 1 | 100.0 | 0 | 0 | 0.0 | 8 | 2 | 25.0 |
| Arunachal Pradesh | 3 | 1 | 33.3 | 10 | 4 | 40.0 | 2 | 0 | 0.0 | 0 | 0 | 0.0 | 1 | 0 | 0.0 | 6 | 0 | 0.0 | 22 | 5 | 22.7 |
| Assam | 18 | 13 | 72.2 | 10 | 7 | 70.0 | 35 | 15 | 42.9 | 45 | 19 | 42.2 | 8 | 6 | 75.0 | 31 | 0 | 0.0 | 147 | 60 | 40.8 |
| Bihar | 3 | 3 | 100.0 | 2 | 2 | 100.0 | 1 | 0 | 0.0 | 2 | 0 | 0.0 | 0 | 0 | 0.0 | 0 | 0 | 0.0 | 8 | 5 | 62.5 |
| Chhattisgarh | 2 | 0 | 0.0 | 4 | 1 | 25.0 | 1 | 0 | 0.0 | 0 | 0 | 0.0 | 1 | 1 | 100.0 | 1 | 1 | 100.0 | 9 | 3 | 33.3 |
| Dadra and Nagar Haveli and Daman and Diu | 0 | 0 | 0.0 | 1 | 1 | 100.0 | 0 | 0 | 0.0 | 0 | 0 | 0.0 | 0 | 0 | 0.0 | 0 | 0 | 0.0 | 1 | 1 | 100.0 |
| Delhi | 0 | 0 | 0.0 | 1 | 1 | 100.0 | 0 | 0 | 0.0 | 0 | 0 | 0.0 | 0 | 0 | 0.0 | 0 | 0 | 0.0 | 1 | 1 | 100.0 |
| Goa | 1 | 1 | 100.0 | 0 | 0 | 0.0 | 0 | 0 | 0.0 | 0 | 0 | 0.0 | 0 | 0 | 0.0 | 0 | 0 | 0.0 | 1 | 1 | 100.0 |
| Gujarat | 2 | 0 | 0.0 | 17 | 11 | 64.7 | 3 | 2 | 66.7 | 1 | 1 | 100.0 | 3 | 2 | 66.7 | 5 | 0 | 0.0 | 31 | 16 | 51.6 |
| Haryana | 0 | 0 | 0.0 | 0 | 0 | 0.0 | 0 | 0 | 0.0 | 1 | 0 | 0.0 | 0 | 0 | 0.0 | 2 | 0 | 0.0 | 3 | 0 | 0.0 |
| Himachal Pradesh | 1 | 0 | 0.0 | 0 | 0 | 0.0 | 0 | 0 | 0.0 | 0 | 0 | 0.0 | 0 | 0 | 0.0 | 0 | 0 | 0.0 | 1 | 0 | 0.0 |
| Jammu And Kashmir | 0 | 0 | 0.0 | 1 | 0 | 0.0 | 0 | 0 | 0.0 | 1 | 1 | 100.0 | 0 | 0 | 0.0 | 1 | 0 | 0.0 | 3 | 1 | 33.3 |
| Jharkhand | 1 | 0 | 0.0 | 3 | 1 | 33.3 | 4 | 2 | 50.0 | 2 | 1 | 50.0 | 1 | 0 | 0.0 | 0 | 0 | 0.0 | 11 | 4 | 36.4 |
| Karnataka | 2 | 1 | 50.0 | 2 | 1 | 50.0 | 2 | 1 | 50.0 | 6 | 2 | 33.3 | 28 | 6 | 21.4 | 30 | 2 | 6.7 | 70 | 13 | 18.6 |
| Kerala | 6 | 2 | 33.3 | 19 | 12 | 63.2 | 2 | 0 | 0.0 | 8 | 5 | 62.5 | 24 | 6 | 25.0 | 17 | 0 | 0.0 | 76 | 25 | 32.9 |
| Madhya Pradesh | 4 | 2 | 50.0 | 1 | 0 | 0.0 | 7 | 0 | 0.0 | 0 | 0 | 0.0 | 5 | 1 | 20.0 | 10 | 1 | 10.0 | 27 | 4 | 14.8 |
| Maharashtra | 10 | 8 | 80.0 | 15 | 9 | 60.0 | 8 | 6 | 75.0 | 9 | 4 | 44.4 | 3 | 1 | 33.3 | 12 | 2 | 16.7 | 57 | 30 | 52.6 |
| Manipur | 9 | 6 | 66.7 | 2 | 0 | 0.0 | 1 | 0 | 0.0 | 0 | 0 | 0.0 | 0 | 0 | 0.0 | 1 | 0 | 0.0 | 13 | 6 | 46.2 |
| Meghalaya | 1 | 1 | 100.0 | 2 | 2 | 100.0 | 1 | 1 | 100.0 | 0 | 0 | 0.0 | 0 | 0 | 0.0 | 6 | 0 | 0.0 | 10 | 4 | 40.0 |
| Mizoram | 4 | 1 | 25.0 | 3 | 2 | 66.7 | 0 | 0 | 0.0 | 0 | 0 | 0.0 | 2 | 2 | 100.0 | 2 | 0 | 0.0 | 11 | 5 | 45.5 |
| Nagaland | 0 | 0 | 0.0 | 2 | 0 | 0.0 | 1 | 0 | 0.0 | 0 | 0 | 0.0 | 0 | 0 | 0.0 | 1 | 0 | 0.0 | 4 | 0 | 0.0 |
| Odisha | 1 | 0 | 0.0 | 5 | 2 | 40.0 | 1 | 0 | 0.0 | 2 | 0 | 0.0 | 0 | 0 | 0.0 | 4 | 0 | 0.0 | 13 | 2 | 15.4 |
| Puducherry | 4 | 3 | 75.0 | 0 | 0 | 0.0 | 2 | 0 | 0.0 | 0 | 0 | 0.0 | 0 | 0 | 0.0 | 0 | 0 | 0.0 | 6 | 3 | 50.0 |
| Punjab | 0 | 0 | 0.0 | 0 | 0 | 0.0 | 0 | 0 | 0.0 | 0 | 0 | 0.0 | 1 | 1 | 100.0 | 0 | 0 | 0.0 | 1 | 1 | 100.0 |
| Rajasthan | 1 | 0 | 0.0 | 3 | 1 | 33.3 | 0 | 0 | 0.0 | 0 | 0 | 0.0 | 0 | 0 | 0.0 | 1 | 0 | 0.0 | 5 | 1 | 20.0 |
| Tamil Nadu | 6 | 1 | 16.7 | 10 | 2 | 20.0 | 0 | 0 | 0.0 | 3 | 1 | 33.3 | 3 | 0 | 0.0 | 3 | 0 | 0.0 | 25 | 4 | 16.0 |
| Tripura | 1 | 0 | 0.0 | 1 | 1 | 100.0 | 0 | 0 | 0.0 | 0 | 0 | 0.0 | 0 | 0 | 0.0 | 0 | 0 | 0.0 | 2 | 1 | 50.0 |
| Uttar Pradesh | 0 | 0 | 0.0 | 0 | 0 | 0.0 | 0 | 0 | 0.0 | 6 | 2 | 33.3 | 3 | 1 | 33.3 | 2 | 1 | 50.0 | 11 | 4 | 36.4 |
| Uttarakhand | 1 | 0 | 0.0 | 0 | 0 | 0.0 | 0 | 0 | 0.0 | 1 | 0 | 0.0 | 1 | 0 | 0.0 | 0 | 0 | 0.0 | 3 | 0 | 0.0 |
| West Bengal | 2 | 0 | 0.0 | 0 | 0 | 0.0 | 1 | 0 | 0.0 | 0 | 0 | 0.0 | 0 | 0 | 0.0 | 0 | 0 | 0.0 | 3 | 0 | 0.0 |
| **Grand Total** | 87 | 43 | 49.4 | 116 | 61 | 52.6 | 72 | 27 | 37.5 | 88 | 36 | 40.9 | 85 | 28 | 32.9 | 135 | 7 | 5.2 | 583 | 202 | 34.6 |

**Supplementary table 4: Distribution of reported zoonotic disease outbreaks by disease type and geographical regions, India, 2018-23**

| **Disease** | **Central** | | | **Eastern** | | | **Northeast** | | | **Northern** | | | **Southern** | | | **Western** | | | **Total** | |
| --- | --- | --- | --- | --- | --- | --- | --- | --- | --- | --- | --- | --- | --- | --- | --- | --- | --- | --- | --- | --- |
|  | **Number of outbreaks** | **Row %** | **Column %** | **Number of outbreaks** | **Row %** | **Column %** | **Number of outbreaks** | **Row %** | **Column %** | **Number of outbreaks** | **Row %** | **Column %** | **Number of outbreaks** | **Row %** | **Column %** | **Number of outbreaks** | **Row %** | **Column %** | **Total outbreaks** | **Column %** |
| Anthrax | 0 | 0.0 | 0.0 | 10 | 62.5 | 28.6 | 0 | 0.0 | 0.0 | 0 | 0.0 | 0.0 | 6 | 37.5 | 3.2 | 0 | 0.0 | 0.0 | 16 | 2.7 |
| Brucellosis | 0 | 0.0 | 0.0 | 0 | 0.0 | 0.0 | 0 | 0.0 | 0.0 | 2 | 33.3 | 11.8 | 4 | 66.7 | 2.2 | 0 | 0.0 | 0.0 | 6 | 1.0 |
| Crimean-Congo Haemorrhagic Fever | 0 | 0.0 | 0.0 | 0 | 0.0 | 0.0 | 0 | 0.0 | 0.0 | 1 | 3.0 | 5.9 | 1 | 3.0 | 0.5 | 31 | 93.9 | 34.4 | 33 | 5.7 |
| Hand Foot Mouth Disease | 0 | 0.0 | 0.0 | 1 | 14.3 | 2.9 | 0 | 0.0 | 0.0 | 2 | 28.6 | 11.8 | 1 | 14.3 | 0.5 | 3 | 42.9 | 3.3 | 7 | 1.2 |
| Influenza | 1 | 33.3 | 2.1 | 0 | 0.0 | 0.0 | 0 | 0.0 | 0.0 | 1 | 33.3 | 5.9 | 1 | 33.3 | 0.5 | 0 | 0.0 | 0.0 | 3 | 0.5 |
| Japanese Encephalitis | 11 | 6.4 | 23.4 | 16 | 9.3 | 45.7 | 115 | 66.9 | 55.0 | 5 | 2.9 | 29.4 | 10 | 5.8 | 5.4 | 15 | 8.7 | 16.7 | 172 | 29.5 |
| Kala Azar | 0 | 0.0 | 0.0 | 4 | 25.0 | 11.4 | 1 | 6.3 | 0.5 | 0 | 0.0 | 0.0 | 11 | 68.8 | 5.9 | 0 | 0.0 | 0.0 | 16 | 2.7 |
| Kyasanur Forest Disease | 0 | 0.0 | 0.0 | 0 | 0.0 | 0.0 | 0 | 0.0 | 0.0 | 0 | 0.0 | 0.0 | 9 | 69.2 | 4.9 | 4 | 30.8 | 4.4 | 13 | 2.2 |
| Leptospirosis | 14 | 12.8 | 29.8 | 0 | 0.0 | 0.0 | 23 | 21.1 | 11.0 | 1 | 0.9 | 5.9 | 49 | 45.0 | 26.5 | 22 | 20.2 | 24.4 | 109 | 18.7 |
| Leptospirosis & Scrub typhus | 1 | 33.3 | 2.1 | 0 | 0.0 | 0.0 | 1 | 33.3 | 0.5 | 0 | 0.0 | 0.0 | 0 | 0.0 | 0.0 | 1 | 33.3 | 1.1 | 3 | 0.5 |
| Melioidosis | 0 | 0.0 | 0.0 | 0 | 0.0 | 0.0 | 0 | 0.0 | 0.0 | 0 | 0.0 | 0.0 | 4 | 80.0 | 2.2 | 1 | 20.0 | 1.1 | 5 | 0.9 |
| Monkey Pox | 0 | 0.0 | 0.0 | 0 | 0.0 | 0.0 | 0 | 0.0 | 0.0 | 0 | 0.0 | 0.0 | 11 | 100.0 | 5.9 | 0 | 0.0 | 0.0 | 11 | 1.9 |
| Nipah | 0 | 0.0 | 0.0 | 0 | 0.0 | 0.0 | 0 | 0.0 | 0.0 | 0 | 0.0 | 0.0 | 2 | 100.0 | 1.1 | 0 | 0.0 | 0.0 | 2 | 0.3 |
| Noro Virus | 0 | 0.0 | 0.0 | 0 | 0.0 | 0.0 | 0 | 0.0 | 0.0 | 0 | 0.0 | 0.0 | 4 | 100.0 | 2.2 | 0 | 0.0 | 0.0 | 4 | 0.7 |
| Rabies | 0 | 0.0 | 0.0 | 0 | 0.0 | 0.0 | 27 | 43.5 | 12.9 | 1 | 1.6 | 5.9 | 34 | 54.8 | 18.4 | 0 | 0.0 | 0.0 | 62 | 10.6 |
| Scrub Typhus | 15 | 18.5 | 31.9 | 4 | 4.9 | 11.4 | 39 | 48.1 | 18.7 | 2 | 2.5 | 11.8 | 11 | 13.6 | 5.9 | 10 | 12.3 | 11.1 | 81 | 13.9 |
| Snake bite | 0 | 0.0 | 0.0 | 0 | 0.0 | 0.0 | 1 | 100.0 | 0.5 | 0 | 0.0 | 0.0 | 0 | 0.0 | 0.0 | 0 | 0.0 | 0.0 | 1 | 0.2 |
| Trypanosomiasis | 0 | 0.0 | 0.0 | 0 | 0.0 | 0.0 | 0 | 0.0 | 0.0 | 1 | 100.0 | 5.9 | 0 | 0.0 | 0.0 | 0 | 0.0 | 0.0 | 1 | 0.2 |
| West Nile Fever | 0 | 0.0 | 0.0 | 0 | 0.0 | 0.0 | 2 | 11.8 | 1.0 | 0 | 0.0 | 0.0 | 15 | 88.2 | 8.1 | 0 | 0.0 | 0.0 | 17 | 2.9 |
| Zika Virus | 5 | 23.8 | 10.6 | 0 | 0.0 | 0.0 | 0 | 0.0 | 0.0 | 1 | 4.8 | 5.9 | 12 | 57.1 | 6.5 | 3 | 14.3 | 3.3 | 21 | 3.6 |
| **Grand Total** | **47** | 8.1 | 100.0 | **35** | 6.0 | 100.0 | **209** | 35.8 | 100.0 | **17** | 2.9 | 100.0 | **185** | 31.7 | 100.0 | **90** | 15.4 | 100.0 | **583** | 100.0 |

**Supplementary table 5: Distribution of zoonotic disease outbreaks (OB) reported late by disease type and geographical regions, India, 2018-23**

| **Disease** | **Central** | | | **Eastern** | | | **Northeast** | | | **Northern** | | | **Southern** | | | **Western** | | |
| --- | --- | --- | --- | --- | --- | --- | --- | --- | --- | --- | --- | --- | --- | --- | --- | --- | --- | --- |
|  | **No. OB** | **No. OB Reported late** | **%** | **No. OB** | **No. OB Reported late** | **%** | **No. OB** | **No. OB Reported late** | **%** | **No. OB** | **No. OB Reported late** | **%** | **No. OB** | **No. OB Reported late** | **%** | **No. OB** | **No. OB Reported late** | **%** |
| Anthrax | 0 | 0 | 0.0 | 10 | 2 | 20.0 | 0 | 0 | 0.0 | 0 | 0 | 0.0 | 6 | 1 | 16.7 | 0 | 0 | 0.0 |
| Brucellosis | 0 | 0 | 0.0 | 0 | 0 | 0.0 | 0 | 0 | 0.0 | 2 | 1 | 50.0 | 4 | 3 | 75.0 | 0 | 0 | 0.0 |
| Crimean-Congo Haemorrhagic Fever | 0 | 0 | 0.0 | 0 | 0 | 0.0 | 0 | 0 | 0.0 | 1 | 0 | 0.0 | 1 | 0 | 0.0 | 31 | 16 | 51.6 |
| Hand Foot Mouth Disease | 0 | 0 | 0.0 | 1 | 0 | 0.0 | 0 | 0 | 0.0 | 2 | 1 | 50.0 | 1 | 0 | 0.0 | 3 | 0 | 0.0 |
| Influenza | 1 | 0 | 0.0 | 0 | 0 | 0.0 | 0 | 0 | 0.0 | 1 | 1 | 100.0 | 1 | 0 | 0.0 | 0 | 0 | 0.0 |
| Japanese Encephalitis | 11 | 4 | 36.4 | 16 | 6 | 37.5 | 115 | 54 | 47.0 | 5 | 0 | 0.0 | 10 | 2 | 20.0 | 15 | 10 | 66.7 |
| Kala Azar | 0 | 0 | 0.0 | 4 | 3 | 75.0 | 1 | 1 | 100.0 | 0 | 0 | 0.0 | 11 | 3 | 27.3 | 0 | 0 | 0.0 |
| Kyasanur Forest Disease | 0 | 0 | 0.0 | 0 | 0 | 0.0 | 0 | 0 | 0.0 | 0 | 0 | 0.0 | 9 | 4 | 44.4 | 4 | 2 | 50.0 |
| Leptospirosis | 14 | 4 | 28.6 | 0 | 0 | 0.0 | 23 | 11 | 47.8 | 1 | 0 | 0.0 | 49 | 9 | 18.4 | 22 | 14 | 63.6 |
| Leptospirosis & Scrub typhus | 1 | 1 | 100.0 | 0 | 0 | 0.0 | 1 | 0 | 0.0 | 0 | 0 | 0.0 | 0 | 0 | 0.0 | 1 | 0 | 0.0 |
| Melioidosis | 0 | 0 | 0.0 | 0 | 0 | 0.0 | 0 | 0 | 0.0 | 0 | 0 | 0.0 | 4 | 2 | 50.0 | 1 | 1 | 100.0 |
| Monkey Pox | 0 | 0 | 0.0 | 0 | 0 | 0.0 | 0 | 0 | 0.0 | 0 | 0 | 0.0 | 11 | 0 | 0.0 | 0 | 0 | 0.0 |
| Nipah | 0 | 0 | 0.0 | 0 | 0 | 0.0 | 0 | 0 | 0.0 | 0 | 0 | 0.0 | 2 | 1 | 50.0 | 0 | 0 | 0.0 |
| Noro Virus | 0 | 0 | 0.0 | 0 | 0 | 0.0 | 0 | 0 | 0.0 | 0 | 0 | 0.0 | 4 | 1 | 25.0 | 0 | 0 | 0.0 |
| Rabies | 0 | 0 | 0.0 | 0 | 0 | 0.0 | 27 | 1 | 3.7 | 1 | 0 | 0.0 | 34 | 5 | 14.7 | 0 | 0 | 0.0 |
| Scrub Typhus | 15 | 2 | 13.3 | 4 | 0 | 0.0 | 39 | 12 | 30.8 | 2 | 0 | 0.0 | 11 | 6 | 54.5 | 10 | 5 | 50.0 |
| Snake bite | 0 | 0 | 0.0 | 0 | 0 | 0.0 | 1 | 0 | 0.0 | 0 | 0 | 0.0 | 0 | 0 | 0.0 | 0 | 0 | 0.0 |
| Trypanosomiasis | 0 | 0 | 0.0 | 0 | 0 | 0.0 | 0 | 0 | 0.0 | 1 | 1 | 100.0 | 0 | 0 | 0.0 | 0 | 0 | 0.0 |
| West Nile Fever | 0 | 0 | 0.0 | 0 | 0 | 0.0 | 2 | 2 | 100.0 | 0 | 0 | 0.0 | 15 | 8 | 53.3 | 0 | 0 | 0.0 |
| Zika Virus | 5 | 0 | 0.0 | 0 | 0 | 0.0 | 0 | 0 | 0.0 | 1 | 0 | 0.0 | 12 | 2 | 16.7 | 3 | 0 | 0.0 |
| **Grand Total** | 47 | 11 | 23.4 | 35 | 11 | 31.4 | 209 | 81 | 38.8 | 17 | 4 | 23.5 | 185 | 47 | 25.4 | 90 | 48 | 53.3 |
